# Magnetic resonance microscopy for submillimeter samples in a horizontal MR scanner

**DOI:** 10.1038/s41598-024-73271-5

**Published:** 2024-10-09

**Authors:** Thomas Hüfken, Tobias Lobmeyer, Bernd Gahr, Fabian Bschorr, Tobias Speidel, Steffen Just, Volker Rasche

**Affiliations:** 1 Department of internal Medicine II, Ulm University Medical Center, Ulm, Germany; 2 Core Facility Small Animal Imaging, Ulm University, Ulm, Germany

**Keywords:** Imaging techniques, Biomedical engineering

## Abstract

The spatial resolution in magnetic resonance imaging is mainly limited by low SNR, which is commonly addressed by long measurement times or dedicated hardware. In single digit micron resolutions, diffusion becomes a further limiting factor since depending on the gradient strength, the diffusion length of particles may approach the target resolutions. Spatial resolution improvement has been addressed by microscopy inserts comprising dedicated gradient systems and RF-coils, usually designed for NMR spectrometers that are often equipped with a deuterium lock for field drift compensations. The presented microscopy insert has been designed to provide single-digit micron resolutions on horizontal preclinical imaging systems utilizing their full imaging and user interface capabilities. The incorporated gradient provides an efficiency of 0.135 T/(m∙A) which in combination with the system’s gradient amplifiers yields a maximum of 27 T/m. With the additional low noise amplifier added to the RF-path a three-fold SNR improvement could be achieved for small samples. Furthermore, a modified constant time imaging sequence was introduced to improve the capability of the setup for ultra‐high-resolution imaging demonstrated on zebrafish embryos at different development stages with (9 μm)³ resolution.

## Introduction

Magnetic resonance imaging is a non-invasive and non‐destructive imaging modality for biological systems and represents one of the most often used imaging techniques in medical science. Even though MRI has focused on macroscopic resolutions, its potential to also provide histology‐like details has been shown in ex-vivo translational neuroimaging^1^ or aortic plaque quantification in mouse models^2^. The capability of high-resolution magnetic resonance microscopy (MRM) in intact living systems was first reported in 1986 at 9.5 T field strength for single-cell imaging of ova obtained from *Xenopus laevis* by Aguayo et al.^3^ with outstanding spatial resolution of 10 μm x 13 μm and 250 μm slice thickness.

MRM is not consistently defined in literature. Benveniste and Blackband^1^ considered MRI as MRM in cases the spatial resolution is below 100 μm in at least one direction. Further developments in MRM have pushed the resolution limits up to 1 μm² in-plane at 75 μm slice thicknesses in phantoms filled with hydrocarbon composite oil^4^, isotropic 3 μm of glass fibers in water^5^ and at a temperature of 5 K to isotropic 1.7 μm in a solid glycerol/water mixture with glass beads^6^. In biological samples, MRM was reported with isotropic resolutions of up to 4.7 μm in chemically fixed rat striatum^7^ or (10 μm)³ in human embryo^8^. Embryos of other species like zebrafish have also been examined^9^. The adult zebrafish as established model in biology has recently been imaged to document in vivo scar formation and heart regeneration with an isotropic resolution of 31 μm^10^ or for brain histology with an optimized fixation at isotropic 10 μm^11^.

MRM is often combined with pure phase encoded acquisitions like constant time imaging (CTI) and as such the spatial resolution is mainly limited by diffusion-introduced broadening of the point spread function (PSF) and the inherently low signal to noise ratio (SNR). An efficient way to reduce PSF’s diffusion introduced broadening is the use of stronger gradients to enable short encoding durations. Some publications of MRM did use frequency encoding^3,4,7,12^, where data is acquired while a magnetic gradient field is applied. This leads not only to susceptibility introduced loss of signal but additionally to geometrical distortions that do not occur in pure phase encoding acquisitions^13^. Further, frequency encoding is more affected by the loss of true resolution due to diffusion, since the maximal possible encoding gradient strength is limited by high acquisition bandwidths and related SNR compromises^14^. Similar limitations do not raise in CTI. However, the related long acquisition times require careful consideration of the drift of the main frequency, which can be extracted from the sample’s free induction decay. These limitations are well-known from other types of sensitive experiments like diffusion^15,16^ and spectroscopic^17,18^ measurements.

The linear decrease of SNR with the voxel volumes often demands extreme overall acquisition times, which have been addressed by optimization of RF-coils and the usage of high magnetic field strengths. Those high field magnets usually belong to spectrometers, some of which can compensate for magnetic field drifts using a deuterium lock. An overview of previously achieved resolutions and related techniques is provided in Table 1. Of note, all published work is based on vertical bore NMR‐spectrometers that had been modified for imaging purposes and are not readily available in conventional biomedical imaging labs. Respective microscopy extensions are also offered by the NMR system manufacturers. But to our knowledge, there are no MRM inserts available for horizontal bore preclinical imaging systems.

**Table 1:** Recently reported magnetic resonance microscopy studies sorted by year.

Name of first author	Sample	Spatial Resolution [µm³]	Acquisition Time [h]	Magnet	RF-Coil	Gradient	Sequence Parameters	Year
Aguayo ^3^	ova obtained from *Xenopus laevis*	10 × 13 × 250	32 min	9.5 T 89-mm bore NMR spectrometer	5 mm solenoid	0.2 T/m	2D-SE TE: 16 ms TR: 4s, FOV: 1.7 × 1.3 mm²	1986
Lee^4^	hydrocarbon composite oil (KyounginEnergy Co., Korea)	1 × 1 × 75	56 min	14.1 T 39.7-mm‐bore vertical	0.5 mm solenoid, Q = 54	10 T/m self-build Golay type	2D-SE TE: 5.5 ms TR: 0.8s FOV: (500 μm)²	2001
Ciobanu^23^	polymer beads in water	3.7 × 3.7 × 3.3	30	9 T 101-mm‐bore vertical (Oxford Instruments)	~ 73 μm solenoid	up to 5.8 T/m^24^	CTI with PHAPS (256 echoes), FOV: 237 × 66 × 66 μm³	2002
Weiger^5^	glass fibers in water	3.0³	58	18.8 T	Surface coil (inner diameter: 20 μm)	24.4 T/m self-build bi-planar (max 65 T/m)	gradient spoiled CTI: TR: 100 ms, BW: 1200 Hz (-> *tacq* = 0.83 ms) FOV: 384 × 384 × 192 μm³	2008
Flint^7^	chemically fixed rat striatum	4.7³	22	14.1 T Bruker spectrometer	Surface coil (Bruker, Z76412)	3 T/m	Gradient Echo: TE: 10 ms, TR: 150 ms, BW: 25 kHz, FOV: (0.6 mm)³	2009
Flint^12^	chemically fixed human spinal cord	6.25³	64	14.1 T Bruker spectrometer	Surface coil (Bruker, B6372)		3D-SE: TR: 2s, TE: 12.75 ms avg: 14	2012
Chen^25^	glass beads in DyEDTA-doped glycerol/water at 28 K	2.8³	52	9.38 T 89 mm bore	~ 160 μm solenoid	up to 4 T/m self-build^26^	Pure phase encoding, FOV: 218 × 95 × 95 μm³	2018
Chen^6^	glass beads in glycerol/water at 5 K using dynamic nuclear polarization	1.7³	81	9.38 T 89 mm bore	~ 115 μm solenoid	up to 4 T/m self-build^26^	Pure phase encoding	2022
Makihara^8^	human embryos	10³	282	9.4 T 89 mm (Oxford Instruments)	10.4 mm solenoid	up to 0.8 T/m	SSFP-FID, FOV: (9.2 mm)³, TR: 100 ms, TE: 6 ms	2023

In this contribution, we introduce a dedicated gradient insert for horizontal bore small animal systems. The insert is implemented as a bi-planar gradient design, which has already shown the potential for MRM through higher gradient strengths and shorter rise times^19,20^. To improve the SNR, an additional PCB with a low-noise amplifier (LNA) in direct proximity to the sample-optimized solenoid RF coil^21,22^ was integrated. For imaging, a constant time imaging sequence was modified (MOCTI) to enable a compensation of frequency drifts and susceptibility induced intensity modulations while optimizing SNR. The overall system performance was demonstrated by imaging zebrafish embryos.

## Materials and methods

The designed microscopy insert was tested in an ultra-high field 11.7 T small animal MRI scanner (BioSpec 117/16, Bruker Biospin, Ettlingen, Germany) operating with ParaVison 7 control software and equipped with Copley 256P gradient amplifiers. In standard configuration the incorporated gradient system has a maximum current / voltage of 200 A / 300 V with a minimum rise time of 130 µs.

### Gradient system

The gradient system in an MRI scanner enables spatial encoding of the MR signal. It utilizes the linear dependency of the spins’ Larmor frequency $\:\omega\:\left(\overrightarrow{r}\right)$ on the amplitude of the magnetic flux density $\:B\left(\overrightarrow{r}\right)$. (With the proportionality factor $\:{\upgamma\:}$ (gyromagnetic ratio) the Larmor frequency is given by $\:\omega\:\left(\overrightarrow{r}\right)={\upgamma\:}B\left(\overrightarrow{r}\right)$.) Under the boundary condition of a homogeneous background magnetic field $\:{\overrightarrow{B}}_{0}$ pointing in z-direction with amplitudes much greater than the magnetic field $\:{\overrightarrow{B}}_{G}$ from the gradient system, the gradient field $\:\overrightarrow{G}$ used for image encoding can be calculated as^27,28^:

1 $$\:\overrightarrow{G}=\:\left.\left(\begin{array}{c}{G}_{x}\\\:{G}_{y}\\\:{G}_{z}\end{array}\right.\right)=\:\left.\left(\begin{array}{c}\frac{\partial\:{B}_{z}}{\partial\:x}\\\:\frac{\partial\:{B}_{z}}{\partial\:y}\\\:\frac{\partial\:{B}_{z}}{\partial\:z}\end{array}\right.\right).$$

Considering an imaged object with a distance of 1 mm from the isocenter, even an amplitude of $\:\overrightarrow{G}$ = 100 T/m results in a $\:{B}_{G}(1\:mm$) = 0.1 T < < 10 T = $\:{B}_{0}$. With varying gradient fields applied, the positions of the magnetic moments can be encoded in frequency domain (k-space). The adjustments of those gradient fields are performed by different currents $\:I$ applied to the gradient system. A metric to describe the ability of the system to generate gradient amplitudes is the gradient efficiency defined as^27^:

2 $$\:\varvec{\eta\:}=\:\frac{\varvec{G}}{\varvec{I}}.$$

### Geometrical optimization

The gradients designed in this work have a bi-planar configuration analog to Weiger et al.^5^ and Da Seeber et al.^25^. For geometrical optimizations, the gradient system was simplified to a configuration of one-dimensional wires as depicted in Fig. 4a, e, i. The rectangular shapes and the current directions in the wires were fixed in the linear model. The *z*-axis of the coordinate system was chosen to be parallel to the main magnetic field $\:{\overrightarrow{B}}_{0}$ (depicted in Fig. 4a, e,i). Variations of the geometrical degrees of freedom *a*_*i*_, *b*_*i*_, *c*_*i*_, and *d*_*x*_ (i $\:\in\:$ {x, y,z}) influence resulting field directions and amplitudes. They are used to maximize the gradient efficiency under the following constraints:

1. An accessible distance between the two planes of 3 mm for positioning of samples and RF-coils. The production relies on double‐sided PCB (four in total). Due to standards in PCB manufacturing processes, the distance to the gradient center is restricted to values of *a*_*i*_ = 1.5, 1.8, 2.1, 2.4 mm. Additionally, the y-gradient must be on one PCB due to the wiring needed for its geometry.
2. The restricted space in the bore of the magnet and the needed width of wires limits the possible values of the geometrical degrees of freedom, which were set to:
  x-gradient: 0.1 mm < *b*_*x*_ < 5 mm, 0.1 mm < *c*_*x*_ < 10 mm, 0.1 mm < *d*_*x*_ < 5 mm.
  y-gradient: 0.1 mm < *b*_*y*_ < 5 mm, 0.1 mm < *c*_*y*_ < 10 mm.
  z-gradient: 0.1 mm < *b*_*z*_ < 10 mm, 0.1 mm < *c*_*z*_ < 10 mm.
3. A cubic volume of (1 mm)³ and a cylindrical volume of 0.8 mm diameter and 1.5 mm length within less than 5% gradient error.

The gradient error at position $\:\overrightarrow{r}$ was calculated as:

3 $$\:{\varvec{E}}_{\varvec{G}}\left(\overrightarrow{\varvec{r}}\right)=\:\frac{{\varvec{B}}_{\varvec{z}}\left(\overrightarrow{\varvec{r}}\right)-\:\frac{\partial\:{\varvec{B}}_{\varvec{z}}\left(0\right)}{\partial\:\overrightarrow{\varvec{r}}}\cdot\:\left|\overrightarrow{\varvec{r}}\right|}{\frac{\partial\:{\varvec{B}}_{\varvec{z}}\left(0\right)}{\partial\:\overrightarrow{\varvec{r}}}\cdot\:\left|\overrightarrow{\varvec{r}}\right|}\:.$$

Calculation of magnetic fields $\:{\overrightarrow{B}}_{G}$ of the linear model was performed utilizing the Biot–Savart law and Wolfram Mathematica 13.2 (Wolfram Research, Champaign, Illinois USA). The calculation of the gradient field $\:\overrightarrow{G}$ was performed according to Eq. (1).

In a first step, maximization of the gradient efficiency was performed for every possible value of *a*_*i*_. Subsequently, the arrangement with values of *a*_*i*_ that maximize the gradient efficiency of the ‘weakest’ direction was chosen.

Gradient strengths and linearity were checked prior to experimental validation by finite element analysis in CST STUDIO SUITE (Dassault Systèmes Simulia Corp., Vélizy-Villacoublay, France) under consideration of a fabricable volume model of the wires.

### Construction

Considering a low installation height of the conductive parts of the gradient system and good heat dissipation the current paths were realized with a PCB (FR4 substrate of 200 μm height, glass transition temperature of 140 °C). The PCB tracks have a height of 35 μm and a minimum width of 1 mm. Isolation of the non-vanished PCBs was achieved by a coating with liquid rubber (Flüssiggummi SPRAY, mibenco gmbh, Karlstein am Main, Germany). PCBs were glued on the holder with a two-part thermal adhesive (Arctic Silver Thermal Adhesive, Arctic Silver Incorporated, Visalia, USA) with negligible electrical conductivity and maximum operation temperature of 150 °C. For correct positioning, four mounting holes in the FR4 substrate were incorporated, exactly fitting the holder pins. Washers added on the pins ensured 3 mm distance in-between the two sides of the gradient system.

Mechanical supply hardware that serves as holders for the PCBs were built of aluminum to ensure mechanical stability and good thermal conductivity. To dissipate heat during a measurement (from inside the magnet’s bore), each side of the holders were constructed to include water-tight fluid channels and connections to water pipes. A thermal sensor (Pt100) allows for temperature monitoring. Its position was determined by the location of the maximum temperature measured with a thermal camera under simultaneous application of currents through all gradients. Electrical connections between the gradients PCBs and the gradient amplifier were realized by attaching ring cable lugs with plastic screws and soldering tin on the blank ends of the conductor paths.

For fixation in the magnet, the aluminum holders were assembled in a u-shaped mounting that is connected to a tube with a clamping mechanism allowing for free rotation of the insert prior to final fixation. For scanning, the PCBs and as such the RF coil were orientated horizontally. The tube can easily be removed with some screws and replaced by another if being used in a larger bore system.

**Fig. 1:**
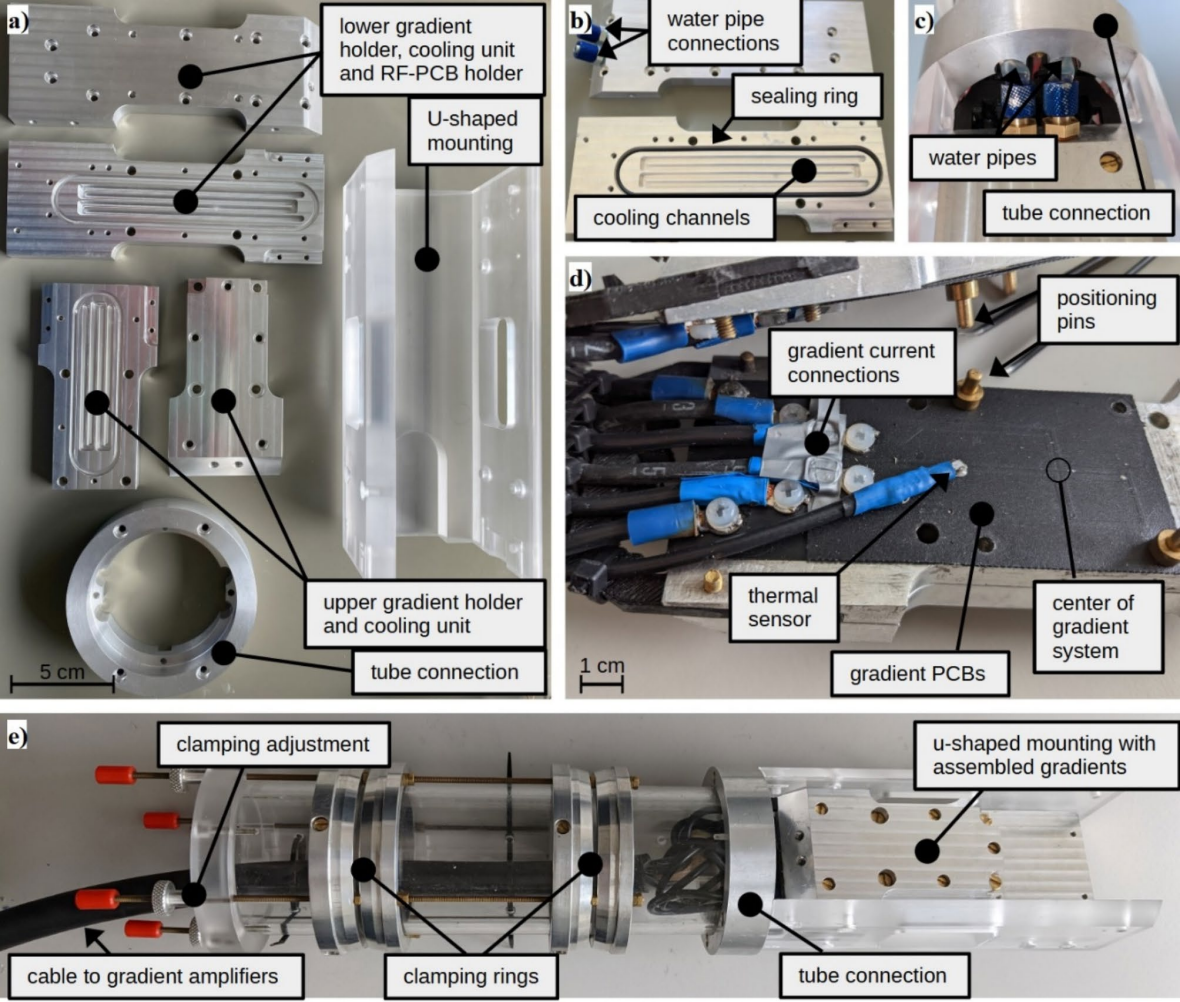
Gradient system and its components. The hardware of the gradient setup before assembling (**a**), lower gradient holder (**b**), connections of the cooling system (**c**), on a holder assembled gradient PCBs (**d**) and the assembled gradient components of the insert including the clamping system (**e**).

An overview of the main components of the gradient system and its respective assembly is provided in Fig. 1.

### Gradient performance tests

The resistance and inductance were measured with an LCR-meter (LCR‐Reader‐MP from Siborg Systems Inc., Ontario, Canada) at 10 kHz and the measurement errors calculated according to the manual.

The gradient efficiency was determined for the x- and z‐direction by direct comparison of the apparent diameter on the MR image and the known diameter of the capillary (see Fig. 7a). The y-direction was derived from the 48 hpf embryo with the dimensions determined by light microscopy (see Fig. 7b-d). The errors of the measured efficiencies in Table 2 were calculated based on the propagation of uncertainty.

Determinations of thermal limits of the gradient system were performed by usage of different currents applied simultaneously to all three gradient channels, and monitoring of the resulting steady-state temperature with the integrated thermal sensor (see positioning in Fig. 1d). The thermal steady-state temperature was assessed for (1) applying a constant current, and (2) a pair of switched trapezoid gradients with opposite polarities played out with a separation of 100 ms, a rise/fall time of 2 ms, and repetition time of 1 s.

### RF-signal‐amplification and Resonator

**Fig. 2:**
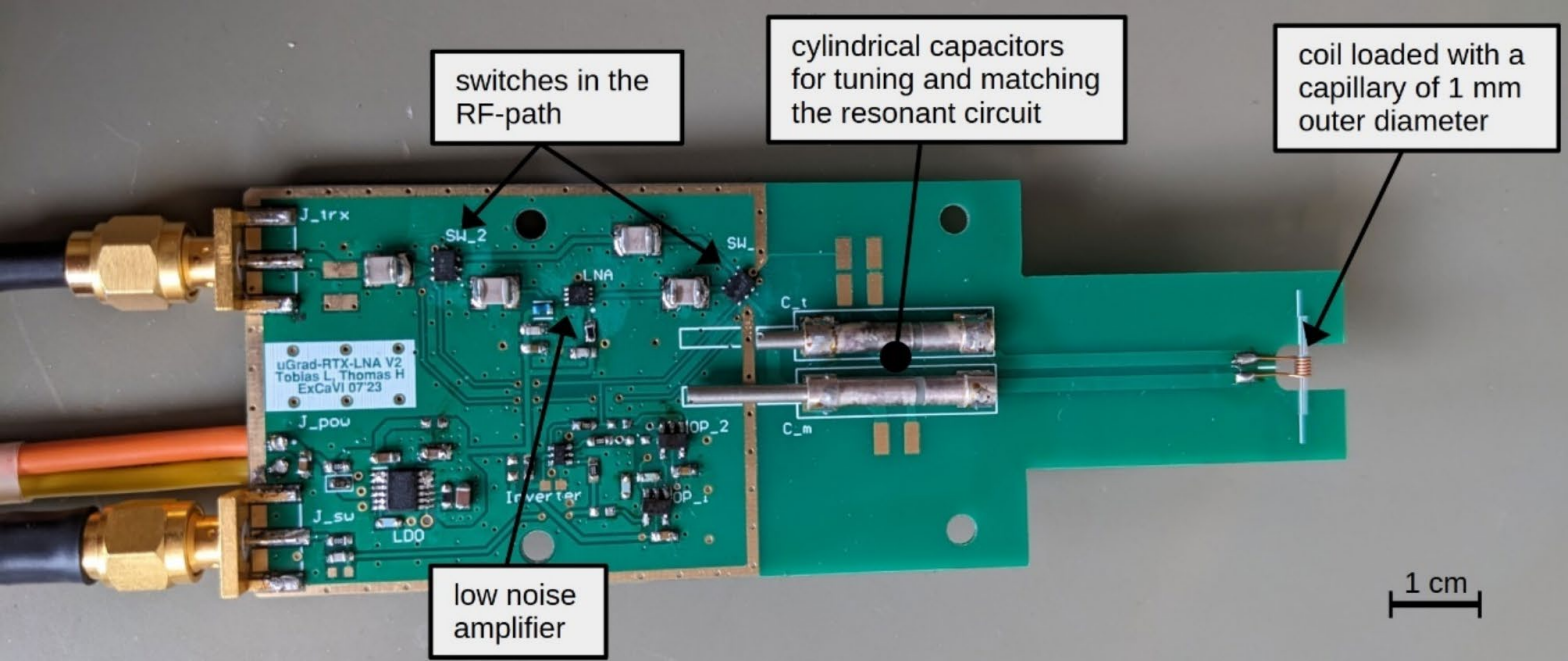
Assembled RF-board with a resonant circuit and a low noise amplifier.

For optimization of the SNR, an additional low noise preamplifier (QPL9547, Qorvo, Greensboro, NC) was added to the RF-chain. A dedicated PCB was designed (Fig. 2) comprising two switches (VSW2‐33–10 W+, Mini‐Circuits, Brooklyn, NY, USA) and the LNA, allowing either for a direct connection of the LC-circuit to the host system in transmit or additional signal amplification in receive mode. The design uses a control voltage of 5 V where the switches have rise and fall times of typically 150 ns and can handle continuous RF power of 10 W for frequencies up to 1 GHz, 9 W RF power up to 2 GHz, and 7 W up to 3 GHz. The combined components enable operations within a frequency range from 100 MHz to 3 GHz. RF-pulses with a peak power of 3 W were successfully tested at 500 MHz. Switching between transmit and receive operation is realized by the blanking signal of the RF‐amplifier. An integrated low-dropout regulator (LDO) ensures a stable power supply for the LNA and switching hardware. For signal reception a 1 mm diameter solenoid coil with 6 turns of 300 μm diameter enameled copper wire is interfaced to the PCB. The required tuning and matching of the resonant circuit are realized by integration of an L‐type network. Further, the proposed PCB includes four screw holes matching the threads of the lower gradient holder for precise integration in the microscopy insert. O‐rings between the PCB and aluminum enable a tilting adjustment. For a sample replacement, the gradient system must be taken out of the magnet and the four screws of the RF-PCB removed.

To compare the performance of the receive path with and without the additional LNA the noise factor (F) defined as the ratio of the input and output SNR was determined with the Y-factor technique^29^, which is based on the determination of two different noise powers ($\:N$) with a setup with equal measurement bandwidths and port impedances. Here, noise powers of a 50 Ohm resistor at room temperature ($\:{T}_{\text{h}\text{o}\text{t}}$ = 294 K) ($\:{N}_{\text{h}\text{o}\text{t}}$) and in liquid nitrogen ($\:{T}_{c\text{o}\text{l}\text{d}}$ = 77 K) ($\:{N}_{\text{c}\text{o}\text{l}\text{d}}$) were measured. The unit-less eponymous Y-factor $\:Y\:=\:{N}_{\text{h}\text{o}\text{t}}/{N}_{\text{c}\text{o}\text{l}\text{d}}$ was derived yielding the effective noise temperature of the connected amplifier (chain) ($\:{T}_{\text{a}\text{m}\text{p}}$) as

4 $${\varvec{T}}_{\varvec{a}\varvec{m}\varvec{p}}\:=\:\frac{{\varvec{T}}_{\varvec{h}\varvec{o}\varvec{t}}\:-\:\varvec{Y}\:\cdot\:{\varvec{T}}_{\varvec{c}\varvec{o}\varvec{l}\varvec{d}}}{\varvec{Y}-1}$$

and the noise figure ($\:NF=10\cdot\:{\text{log}}_{10}\left(F\right)$) corresponding to the reference temperature of $\:{T}_{0}=$ 290 K:

5 $$\:NF=10\cdot\:{log}\left(\frac{{T}_{amp}+{T}_{0}}{{T}_{0}}\right).$$

### Sample preparation

Zebrafish (*Danio rerio*) of the TüAB strain were kept and bred as previously described^30^ according to institutional approvals (Tierforschungszentrum (TFZ) Ulm University, No. z.183) in accordance with EU Directive 2010/63/EU. Embryos were kept in E3 medium (5 mM NaCl, 0.17 mM KCl, 0.33 mM CaCl2, 0.33 mM MgSO4, 0.6 µM Methylene Blue dissolved in water), euthanized in 0.03% Ticain (MS222) and fixated in 4% PA for 2 h at room temperature at 32 and 48 h post fertilization (hpf) and transferred to glass capillaries with an inner diameter of 800 μm (Pyrex NMR Capillary Tubes from SP Wilmad-LabGlass, Vineland, New Jersey, USA). The embryos were embedded in Flourinert (3 M, Maplewood, MN, USA) to reduce susceptibility introduced artifacts. As Fluorinert contains no hydrogen atoms it is invisible in 1 H MRI. Furthermore, it is a liquid that matches the susceptibility of water to reduce the effect of different susceptibilities and increase the measurable signal^31^. The ends of the capillary were sealed with wax to prevent evaporation and the loss of any liquids. Figure 7c, d show a microscopy image of a prepared sample. After the preparation, the sample was transferred into the coil and the ends of the capillary were attached to the PCB with adhesive tape to prevent any movement during the measurement process.

Additional phantoms for assessing the effect of the gradient strength on the image quality were prepared using the same type of capillaries but filled with tap water and sealed with wax.

### Data acquisition for imaging

**Fig. 3:**
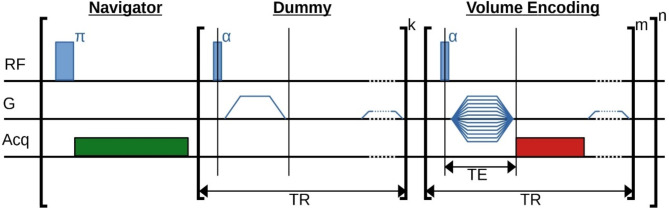
Scheme of the modified constant time imaging (MOCTI) sequence. It demonstrates the timings of the application of the radio frequency pulses (RF), the magnetic gradient fields (G) and acquisition periods (Acq). The green acquisition block acquires data for the correction of a frequency drift (navigator) and the red block for the k-space (spatial encoding). The repetition time (TR) and echo time (TE) are indicated by arrows. Brackets indicate sequence blocks that are repeated as often as indicated by the exponents.

For ultra-high-resolution images, a **mo**dified **c**onstant **t**ime **i**maging sequence (MOCTI) was applied (Fig. 3). It is based on a pure phase encoding sequence that divides the acquisition of the k-space into $\:n$ segments. Each of the $\:m$ k-space points within one segment are encoded by a conventional CTI technique with a trailing spoiler gradient to prevent a pile-up of unwanted signal. For each k-space point, the MR signal is acquired over a certain readout period with sampling bandwidth $\:B{W}_{k}$. Before measuring the k-space data of a single segment, the sample’s resonance frequency (navigator) is derived from a single free induction decay (FID) measurement at sampling bandwidth $\:B{W}_{\text{n}\text{a}\text{v}}$. To ensure steady-state condition during subsequent data acquisitions a leading block of $\:k$ dummy cycles is applied in-between navigator and data acquisition.

To correct for frequency drifts, the measurements of the zebrafish embryos were subdivided into $\:n$ = 64 segments resulting in a frequency determination every 44 min. 2048 data points were acquired for each FID with $\:B{W}_{\text{n}\text{a}\text{v}}$ = 10 kHz within a navigator block of 1 s duration. Measuring k-space was resumed after $\:k$ = 200 dummy cycles. The navigators and dummies caused an overall prolongation of the absolute scan duration by 18 min (0.64%). Further relevant scan parameters were: echo time (TE) = 0.8 ms, repetition time (TR) = 80 ms, flip angle α = 28°, $\:B{W}_{\text{k}}$ = 8 kHz for 512 sample points, resolution: (9 μm)^2^, matrix size: (128)^2^ and total scan time of 46 h 54 min. The maximum gradient strength needed for encoding was 1.5 T/m with a ramp time of 130 µs using a duty cycle of 1.1%. For excitation of the spatially encoded signal, block pulses with a length of 12.8 µs and a transmit power of 0.027 W were used. Prior to the measurements, linear shimming was performed as conventionally available on the imaging system.

For imaging of the water phantom, different gradient amplitudes (0.025, 0.25, 2.5, 5 and 9 T/m) were used to demonstrate the application of higher gradient amplitudes and their influence on echo time and image quality. The matrix of 128 × 128 × 16 was acquired with MOCTI for a resolution of 10 × 10 × 80 μm. The other parameters were: k = 200 dummy cycles, TR = 80 ms, $\:B{W}_{\text{k}}$ = 200 kHz for 2048 sample points, flip angle α = 20° provided by a 1.38 W and 2 µs block pulse, and a total scan time of 5 h 50 min.

### Image reconstruction

The drift of the resonance frequency $\:{\omega\:}_{0}$ is extracted from the navigator signal by a Fourier transformation (FT). A linear extrapolation between subsequent $\:{\omega\:}_{0}$ measurements is used for estimation of the k-space dependent drift of the main frequency $\:{\omega\:}_{0}\left(\overrightarrow{k}\right)$. To correct for this unwanted drift, the acquired complex signal $\:{s}_{\text{a}\text{c}\text{q}}\left(\overrightarrow{k},{t}_{\text{a}\text{c}\text{q}}\right)$ is corrected by:

6 $$\:{s}_{{\omega\:}_{0}\_corr}\left(\overrightarrow{k},{t}_{acq}\right)={s}_{acq}\left(\overrightarrow{k},{t}_{acq}\right)\cdot\:\:{e}^{-i\cdot\:{\omega\:}_{0}\left(\overrightarrow{k}\right)\:\cdot\:\:{t}_{acq}}\:$$

with $\:{t}_{\text{a}\text{c}\text{q}}$ being the time of the acquisition of the specific k-space point $\:\overrightarrow{k}$.

In a second step, errors introduced by spatially varying deviations $\:{\Delta\:}B\left(\overrightarrow{r}\right)$ from the main magnetic field $\:{B}_{0}$ (mainly caused, among other effects, by spatial variations in magnetic susceptibility) which are supposed to be time independent, are addressed by deriving a field map $\:{\Delta\:}B\left(\overrightarrow{r}\right)$ from the acquired CTI data. Assuming a smooth variation of $\:{\Delta\:}B\left(\overrightarrow{r}\right)$, for SNR considerations, $\:{\Delta\:}B\left(\overrightarrow{r}\right)$ estimation is performed based on the central 50% of k-space data with additional application of a Gaussian smoothing filter ($\:{s}_{{{\upomega\:}}_{0}\_\text{c}\text{o}\text{r}\text{r},\text{s}\text{m}\text{a}\text{l}\text{l}}$). Low-resolution images $\:{S}_{\text{s}\text{m}\text{a}\text{l}\text{l}}$ covering the temporal evolution of the data over the readout period are derived by inverse FT as $\:{S}_{{{\upomega\:}}_{0}\_\text{c}\text{o}\text{r}\text{r},\text{s}\text{m}\text{a}\text{l}\text{l}}\left(\overrightarrow{r},\:{t}_{\text{a}\text{c}\text{q}}\right)=\:{\mathcal{F}}^{-1}\left\{{s}_{{{\upomega\:}}_{0}\_\text{c}\text{o}\text{r}\text{r},\text{s}\text{m}\text{a}\text{l}\text{l}}(\overrightarrow{k},{t}_{\text{a}\text{c}\text{q}})\right\}$. FT along the time dimension of $\:{S}_{{{\upomega\:}}_{0}\_\text{c}\text{o}\text{r}\text{r},\text{s}\text{m}\text{a}\text{l}\text{l}}(\overrightarrow{r},\:{t}_{\text{a}\text{c}\text{q}})$ yields $\:{\Delta\:}B\left(\overrightarrow{r}\right)$ from the point with maximum amplitude in the spectrum since $\:\omega\:\left(\overrightarrow{r}\right)=\:\gamma\:\cdot\:{\Delta\:}B\left(\overrightarrow{r}\right)$. To exclude noise pixel from the calculations, pixel with peak amplitudes below 10% of the maximum peak amplitude were excluded from correction. In a final step the resulting $\:{\Delta\:}B\left(\overrightarrow{r}\right)$ map is interpolated to full spatial resolution and used for correction of the spatially dependent phase accrual according to:

7 $$\:{S}_{\varDelta\:B\_corr,\:\:{\omega\:}_{0}\_corr}\left(\overrightarrow{r},{t}_{acq}\right)={S}_{{\omega\:}_{0}\_corr}\left(\overrightarrow{r},{t}_{acq}\right)\cdot\:{e}^{-i\cdot\:\gamma\:\cdot\:\varDelta\:{B}_{0}\left(\overrightarrow{r}\right)\cdot\:{t}_{acq}}$$.

The final image is calculated by averaging $\:{S}_{\varDelta\:B\_\text{c}\text{o}\text{r}\text{r},\:\:{{\upomega\:}}_{0}\_\text{c}\text{o}\text{r}\text{r}}\left(\overrightarrow{r},{t}_{\text{a}\text{c}\text{q}}\right)$ along the time dimension. Imperfect corrections caused by noisy data and resolution limits of the spectra requires optimization of the time frame considered for temporal averaging of the data, which is done by optimization of the contrast-to-noise ratio (CNR) in the final images with a different number of time points used for averaging. Therefore, two regions of interest (ROI) are defined in the images. One ROI is located in pure noise ($\:RO{I}_{\text{n}\text{o}\text{i}\text{s}\text{e}}$) and the other ($\:RO{I}_{\text{s}\text{i}\text{g}\text{n}\text{a}\text{l}}$) in a region of signal important for a later inspection. The CNR was calculated according to:

8 $$\:CNR=\:0.66\cdot\:\frac{mean\left(RO{I}_{signal}\right)-mean\left(\:RO{I}_{noise}\right)\:}{std\left(RO{I}_{noise}\right)}.$$.

The factor of ‘0.66’ in Eq. (8) is needed to correct for the fact that $\:std\left(RO{I}_{\text{n}\text{o}\text{i}\text{s}\text{e}}\right)$ is determined in magnitude images (Rician noise distribution)^32^. A calculation of CNR instead of a classical SNR calculation was chosen because the expected signal amplitudes are small and due to inspection of magnitude images there is a not negligible mean value > 0 in regions of pure noise.

### True resolution

Since the point spread function (PSF) of imaging with MOCTI is a convolution of sampling and diffusion affected PSFs there is no analytical expression for the final full width at half maximum (FWHM), and simulations are needed. A proper analytical function estimating the relative loss of resolution in MRM is given by Weiger et al.^5^. It is well applicable for ranges of diffusion coefficients $\:D$ = 0.15–4.8 μm²/ms, measured spatial resolutions $\:\varDelta\:$ = 1–10 μm and gradient strengths $\:G$ = 0.01–104 T/m. In terms of truly inspectable resolution $\:{\varDelta\:}_{\text{t}\text{r}\text{u}\text{e}}$ it can be expressed as:

9 $$\:{\varDelta\:}_{true}=1.21\cdot\:\varDelta\:\cdot\:\left(1+{\left(\frac{G}{{G}_{0}}\right)}^{\raisebox{1ex}{$1$}\!\left/\:\!\raisebox{-1ex}{$a$}\right.}\cdot\:{\left(\frac{{D}_{0}}{D}\right)}^{\raisebox{1ex}{$1$}\!\left/\:\!\raisebox{-1ex}{$a$}\right.}\cdot\:{\left(\frac{\varDelta\:}{{\varDelta\:}_{0}}\right)}^{\raisebox{1ex}{$3$}\!\left/\:\!\raisebox{-1ex}{$a$}\right.}\right)$$

with constants $\:{\text{D}}_{0}$ = 1 μm²/ms, $\:{\text{G}}_{0}$ = 7.37 T/m, $\:{\varDelta\:}_{0}$ = 1 μm, and $\:\text{a}$ = − 0.938.

## Results

### Gradient geometry

**Fig. 4:**
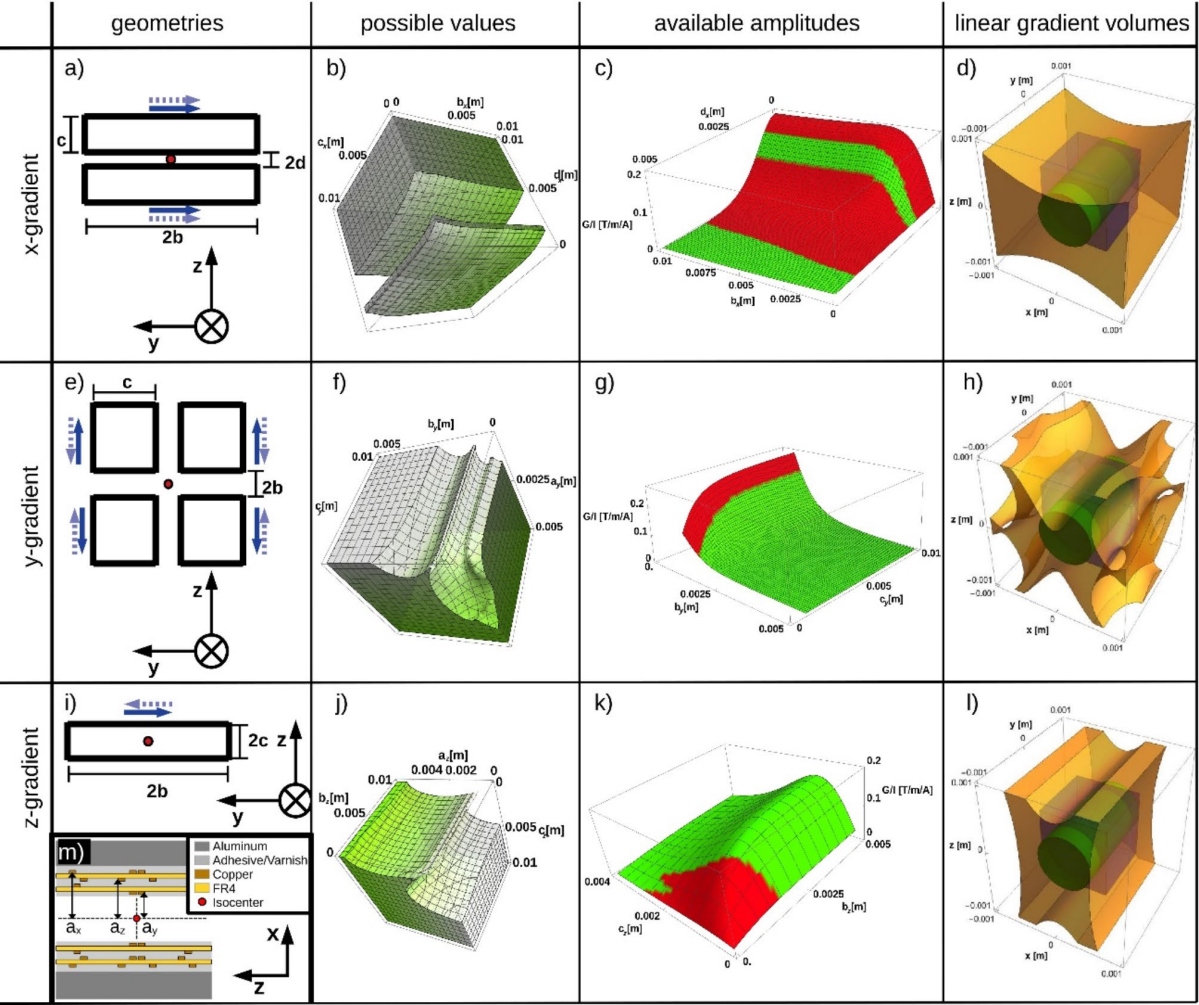
Geometrical optimization of the gradients in x- (a, b, c, d), y‐ (e, f, g, h) and z‐direction (i, j, k, l). Geometries with their degrees of freedom ‘b_i_’, ‘c_i_’ and ‘d_i_’ are depicted in black (a, e, i). Distances ‘a_i_’ of the parallel planes to the origin (red dot) in m). Current directions in one plane are symbolized as dotted and in the second plane as plain arrows. Possible values of the geometrical degrees of freedom that fulfill the gradient linearity constraints in the regions of interest are within the volumes presented in the second column. Gradient amplitudes (green: linearity satisfied, red: insufficient linearity) are in c), g) and k). Volumes of the final geometry that fulfill linearity of the optimized system are depicted in yellow in d), h) and l) and include the regions of interest (blue cube and green cylinder).

Possible values for the geometry’s variables *a*_*i*_, *b*_*i*_, *c*_*i*_ and *d*_*x*_ that provide $\:{E}_{G}\left(\overrightarrow{r}\right)<0.05$ in the regions of interest (cube and cylinder) are depicted in the second column of Fig. 4. Gradient strengths of geometrical combinations are demonstrated in the third column.

The optimization of the gradient system resulted in the following geometrical values: for the x-gradient: a_x_ = 2.1 mm, b_x_ = 5 mm, c_x_ = 10 mm, and d_x_ = 0.75 mm; for the y‐gradient: a_y_ = 1.5 mm, b_y_ = 0.75 mm, and c_y_ = 10 mm; z‐gradient: a_z_ = 1.8 mm, b_z_ = 10 mm, and c_z_ = 1.5 mm.

The final spatial regions with sufficient gradient linearity are shown as yellow volumes in Fig. 4d, h,l. The resulting gradient efficiency values of the calculation with linear wires and of the validation by finite element analysis in CST are presented in Table 2.

### Gradient performance

Characteristic properties of the gradient system are summarized in Table 2.

**Table 2:** Characteristics of the gradient’s performance.

Gradient	Efficiency linear model [T/(m A)]	Efficiency CST [T/(m A)]	Efficiency measured [T/(m A)]	Resistance [Ohm]	Inductance [µH]
X-Gradient	0.138	0.123	0.135 ± 0.008	0.08 ± 0.02	0.78 ± 0.07
Y-Gradient	0.192	0.173	0.167 ± 0.04	0.10 ± 0.03	0.83 ± 0.07
Z-Gradient	0.143	0.125	0.137 ± 0.008	0.51 ± 0.05	0.71 ± 0.07

**Fig. 5:**
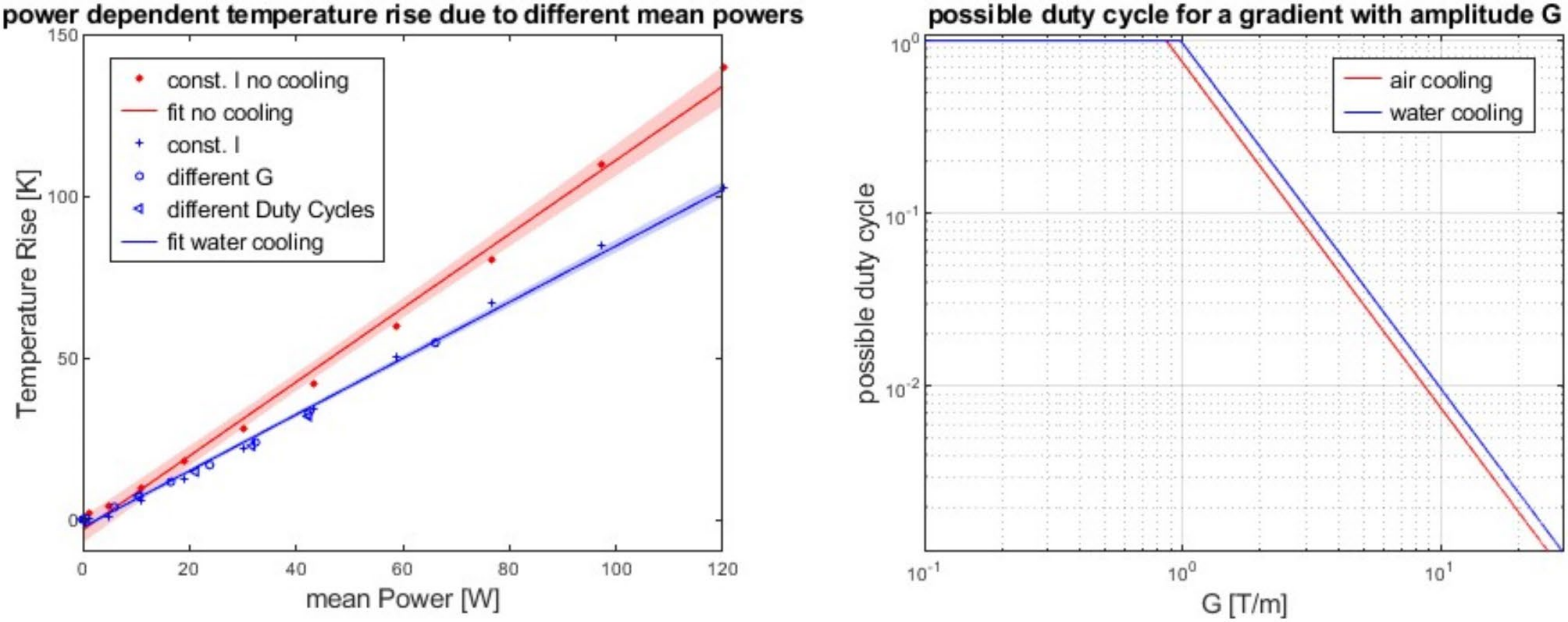
Temperature rise of the system due to different mean powers (left). Data is grouped in with (blue) and without (red) water cooling. Possible duty cycles for a given gradient amplitude and a maximum temperature rise of 50 K (right).

Figure 5 demonstrates the temperature dependent performance of the gradient system with (blue) and without (red) active water cooling. The (quasi-)steady state temperature was reached within a few seconds for all measurements. Measurements of increasing constant currents did show a quadratic dependency of the temperature rise on the currents (cross and star), indicating linear dependency on the electrical power. A similar power dependency was observed for bipolar gradient pairs. Confirmation measurements were performed with constant duty cycle of 20% with gradient amplitudes of 0.27, 0.83, 1.1, 1.4, 1.6, 1.9, 2.2, and 2.7 T/m on every gradient direction played out simultaneously and with a constant amplitude of 1.1 T/m and different effective duty cycles of 20, 40, 60, and 80%. Linear fits to data of the active and passive setups are depicted as well as the fit’s 95% confidence intervals. The measured temperature rises for the system resulted to 0.870 K/W with and 1.143 K/W without active cooling. The resulting possible duty cycles for a given gradient amplitude considering a maximum temperature rise of 50 K is depicted in the right graph of Fig. 5.

### Receiver improvement

Noise figures of the RF-PCB were determined using a 50 Ohm load connected to the corresponding interface on the PCB. Three different settings were analyzed: thermal load connected to the PCB and direct path to spectrometer (transmit mode), thermal load connected to the PCB and signal amplified with LNA (receive mode) and thermal load directly connected to the vendor’s hardware (no RF-PCB). The results are summarized in Table 3.

**Table 3:** Noise factors and noise figures of the different receive paths tested.

Setup	Noise factor	Noise figure [dB]
RF-PCB in receive mode	1.28	1.09
RF-PCB in transmit mode (no LNA)	3.03	4.81
Direct connection (no RF-PCB)	4.18	6.21

### Imaging

**Fig. 6:**
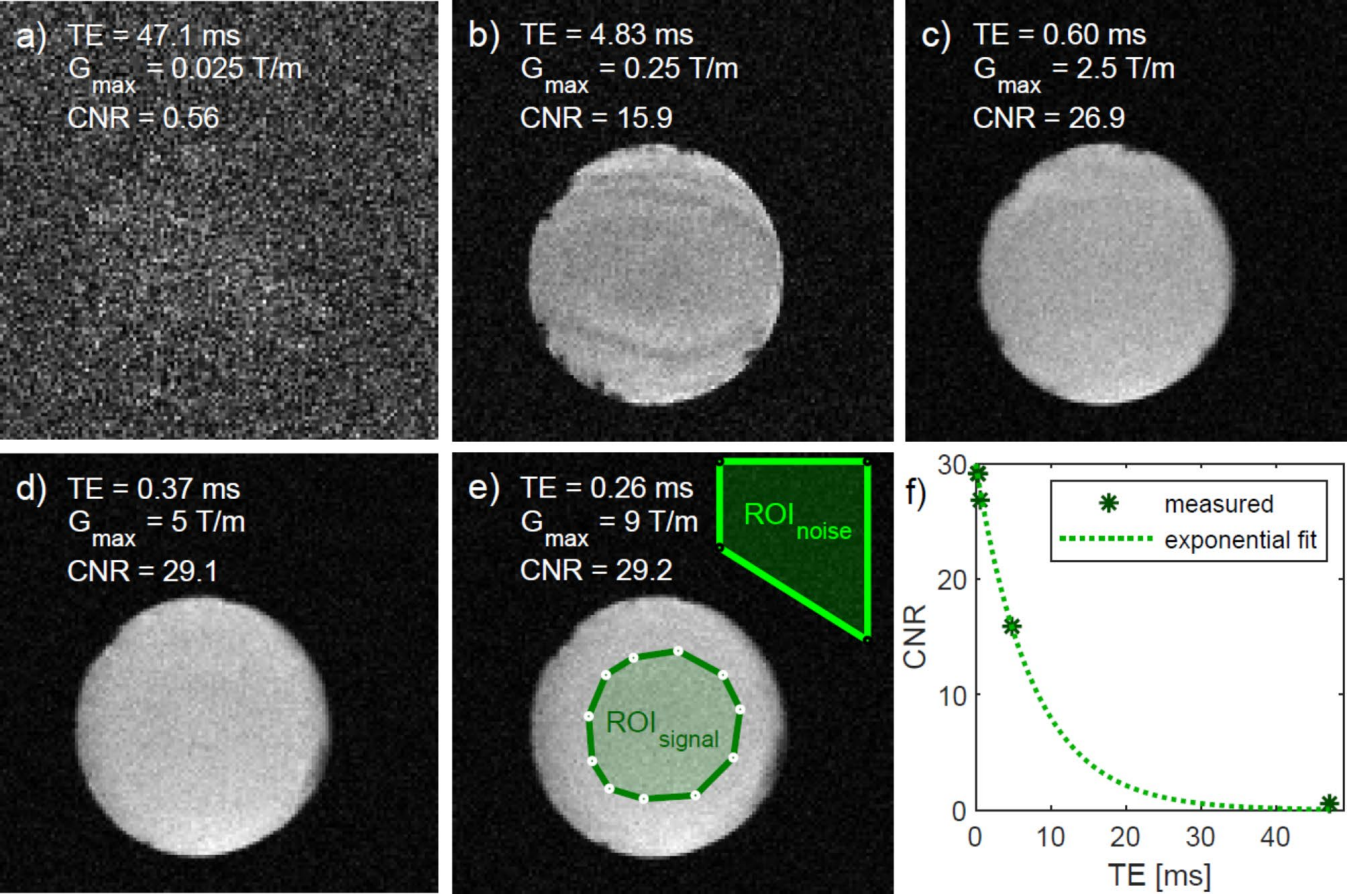
CNR comparison of CTI of a water filled capillary acquired with different gradient amplitudes (a-e). For the calculation of CNRs the ROIs as depicted in e) were used for all images. An exponential decay of CNR with increasing echo time is depicted in f).

**Fig. 7:**
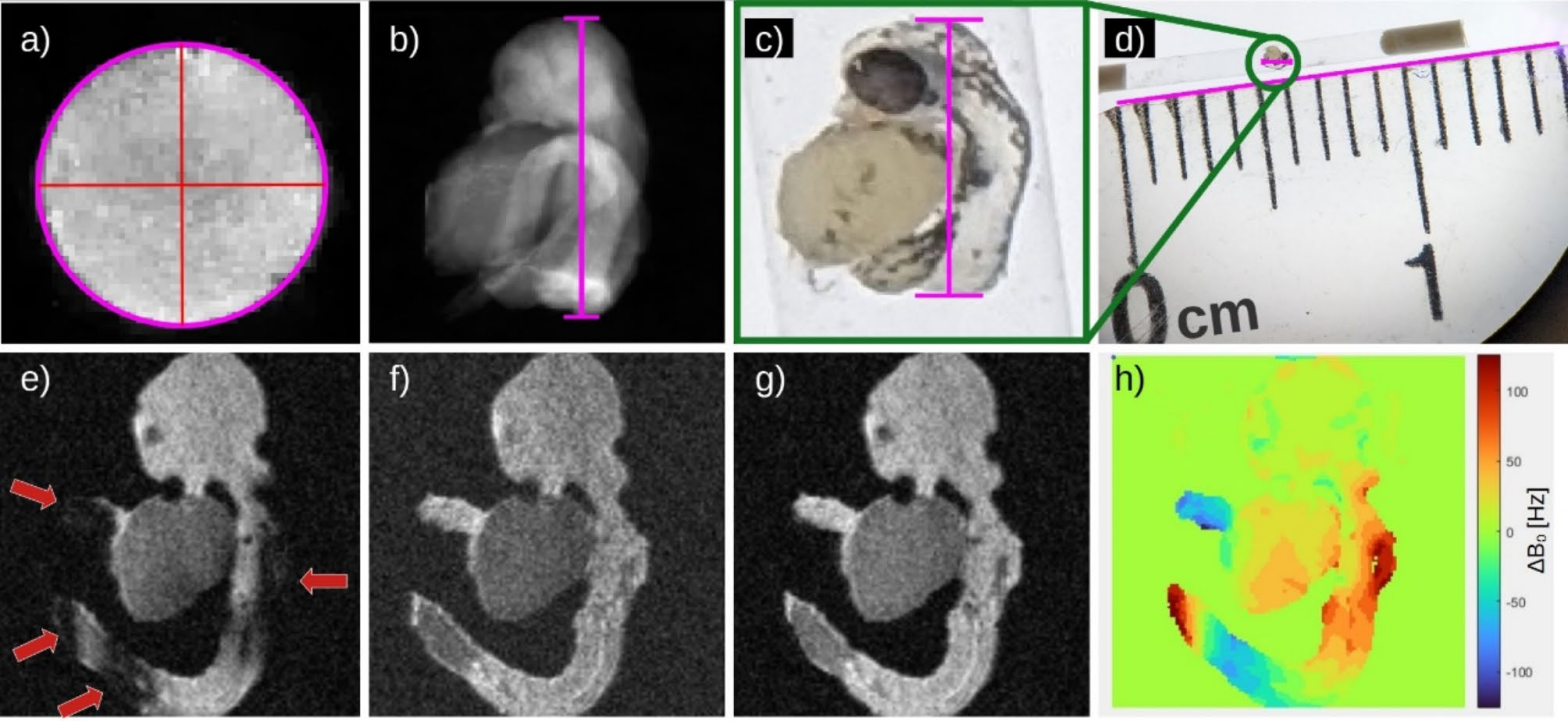
Images to determine gradient and sequence performance. MR image of a capillary filled with water (**a**) including an ellipse fitted to the edge (horizontal axis length: 50.64 pixel, vertical axis: 50.2 pixel) to determine gradient amplitudes. In (**b**) a projection of a zebrafish embryo fixated at 48 hpf is depicted with a scale bare length of 121 pixels corresponding to 0.94 mm as extracted from the respective images from the optical microscope at different scales (**c,d**). Resulting images (**e-g**) using the same data, reconstructed with complex averaging (e), magnitude averaging (**f**) and MOCTI reconstruction (**g**) with the corresponding $\:{\Delta\:}\text{B}\left(\overrightarrow{\text{r}}\right)$-map (**h**). Red arrows in (**e**) indicate incoherent signal summation due to $\:{\Delta\:}\text{B}\left(\overrightarrow{\text{r}}\right)$.

Results obtained with different gradient amplitudes are shown in Fig. 6. Depending on the gradient strength TE resulted to 47.1 ms, 4.83 ms,0.6 ms, 0.37 ms and 0.26 ms. All reconstructions were calculated using 440 time points resulting from a CNR optimization on image e). The analysis of the signal intensities of the images provides an exponential decay dependent on the echo time as depicted in Fig. 6f. Figure 7e-g show images of the same slice reconstructed from the same drift-corrected data using the same 110 time points. This 110 time points correspond to a measurement time of 13.75 ms for a single point in k-space. Figure 7h is the $\:{\Delta\:}{B}_{0}$ map used for image reconstruction of Fig. 7g. Figure 7e shows the result from complex summation. Clear signal voids caused by the phase accrual due to $\:{\Delta\:}B\left(\overrightarrow{r}\right)$ can be inspected. Figure 7f shows the sum of the respective magnitude images resulting in an artifact-free image with rather poor CNR. Substantially improved CNR with no apparent artifacts is obtained by consideration of $\:{\Delta\:}B\left(\overrightarrow{r}\right)$ prior to image summation (Fig. 7g). To compare the effect of the different reconstruction approaches the resulting CNR of the artifact-free images is presented in Table 4, which clearly reveals the advantage of performing a phase-corrected reconstruction under full consideration of the of $\:{\Delta\:}B\left(\overrightarrow{r}\right)$.

**Table 4:** Comparison of the contrast to noise ratio (CNR) of the different reconstruction methods. Non-phase corrected data was excluded due to obvious signal voids and modulations.

Reconstruction method	CNR head	CNR yolk
Addition of magnitude images	7.17	4.29
MOCTI reconstruction	13.01	9.44

**Fig. 8:**
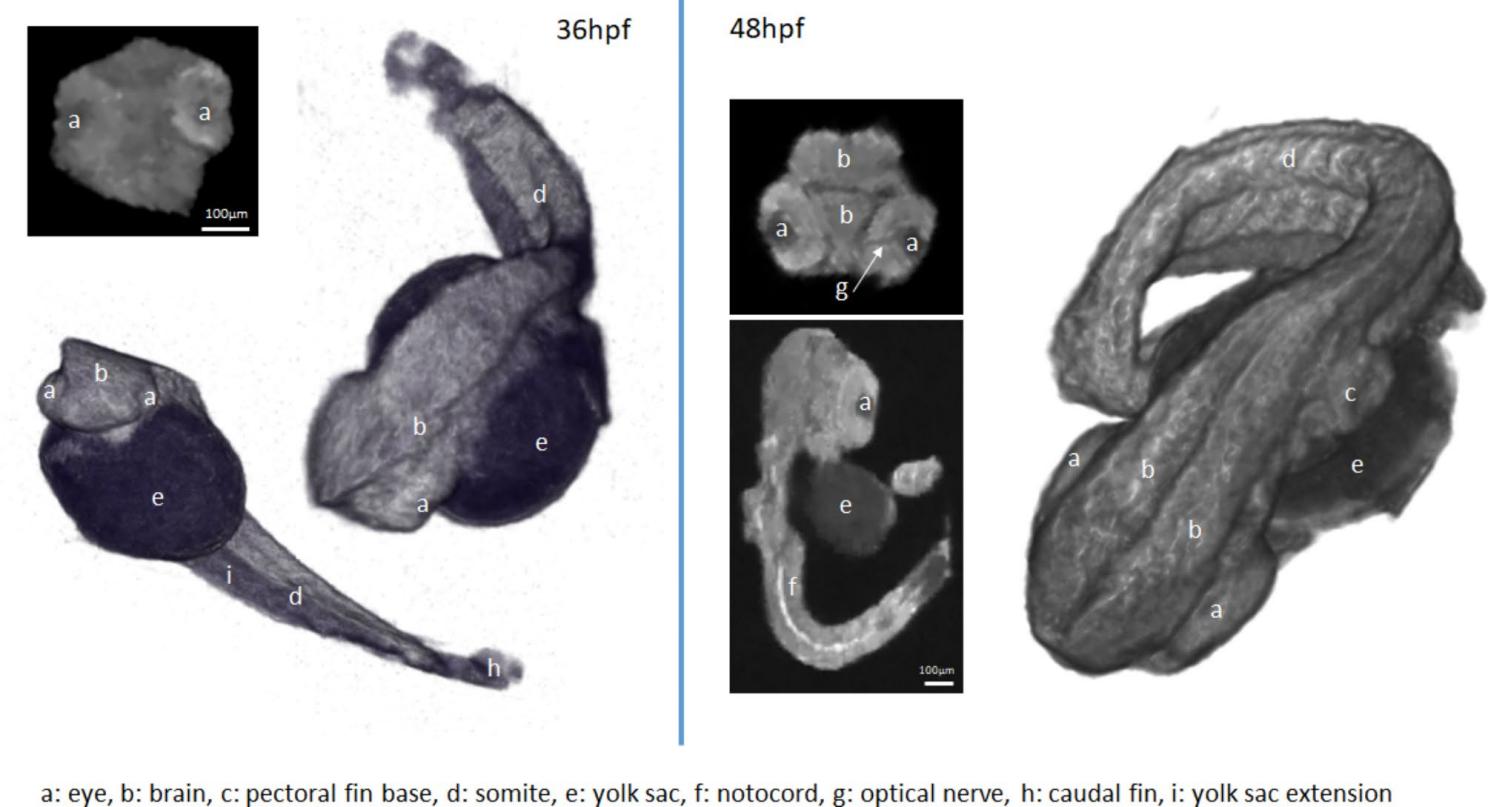
2D slices and volumetric renderings of MR imaged zebrafish embryos fixated at 36 and 48 hpf with labeled anatomy.

Three-dimensional renderings (3D Slicer, www.slicer.org ^33^) and multiplanar reformats (MPR) of the 36 hpf (left) and 48 hpf (right) embryo are shown in Fig. 8. The 3D renderings clearly reveal the development of the embryo and in 48 hpf much more anatomical details can be appreciated. All data presented have been additionally filtered by a total variation (TV) filter for further noise reduction. While still limited by the spatial resolution, even at 36 hpf some anatomical details can be clearly appreciated, which become more apparent at 48 hpf.

Frequency drifts in the order of 100 Hz were observed over the 47 h scan.

Considering the impact of diffusion, the true achieved spatial resolution in the data presented assuming a diffusion coefficient $\:D$ = 2.2 μm²/ms, measured spatial resolutions $\:\varDelta\:$ = 9 μm, and gradient strengths $\:G$ = 1.5 T/m results to $\:{\varDelta\:}_{true}$ = 11.0 μm.

## Discussion

This work demonstrates the feasibility of a microscopy insert for MRM applications in a conventional preclinical horizontal bore scanner. The PCB-based gradient system enables ultra-high resolution imaging for small samples of about (1 mm)³. The high gradient amplitude ensures short echo times and less impact of diffusion introduced image artifacts. The integration of an additional LNA in direct proximity to the small samples further improves the resulting SNR. With the proposed system imaging of zebrafish embryos was shown at ultra-high isotropic resolution of sub 10 μm in combination with the introduced MOCTI sequence.

The measured gradient performance (Table 2) is equal within the scope of measurement accuracy to the values determined by the linear model but in slight disagreement with the CST simulations for the x- and z-gradient. This can likely be explained by the inaccuracy of the assembly process where especially the layer thickness of the thermal adhesive is not perfectly reproducible e.g. causing the wires of the x- and z-gradient (both are located on one PCB) to came closer to the gradient center with related higher gradient amplitudes. This demonstrates that for a gradient linearity based geometrical optimization the simulation of a linear geometry appears sufficient. However, both methods will not provide the absolute gradient efficiencies and considerations of a three-dimensional conductor geometry of the finally assembled system might be needed.

The resistance and inductance of the gradient system are small. According to Stehling et al.^34^ this would enable theoretical rise times of less than 1 µs for a current of 200 A with a voltage of 300 V. This is two orders of magnitude shorter than typical rise times used in MRI. The practical rise time is much higher not only due to the additional wiring from the amplifiers to the scanner room but also because of the control frequencies of gradient amplifiers that are rather in the range of kHz instead of MHz. In the unmodified preclinical system, equipped with Copley 265P amplifiers, the minimum rise time was set to 130 µs, which could likely be reduced for optimal performance to the gradient system. The maximal gradient amplitude of the gradient system is currently limited by the x-gradient and theoretically results to 27 T/m with a duty cycle of about 0.13%. ost other gradient systems used for MRM were reported to have an order of magnitude less amplitude with the exception of the system of Weiger et al.^5^, which should provide an amplitudes of 65 T/m at a duty cycle of 0.4%. To chieve a higher gradient strength, the demonstrated gradient coil design would need more turns thereby causing an almost linear increase in resistance and thus heat.

With the water-cooling system, we could show that a 17% higher power duty cycle is possible. Adding additional thermal sensors will allow for a more precise temperature monitoring of the gradient system and likely further improve the duty-cycle.

Noise factor measurements of the unaltered vendor’s receive path shows a noise factor of 4.18. Adding an additional LNA in direct proximity to the samples revealed a possible three-fold SNR improvement. Due to the vendor’s hardware being a closed system, the dominant noise contributors are unclear. Possible dominant noise sources may be multiple connectors and cables between coil and vendor’s LNA or the LNA itself. Adding an additional LNA reduces the noise contribution of the beforementioned receive path according to Friis’ formula^35^. The gain of performance can be used for a reduction of scan time by a factor of 9 or a two-fold improvement of the spatial resolution. The observed lower noise figure of the receive path including the PCB in transmit mode (no LNA included) compared to the classical scanner path might be caused by parasitic gains introduced by the LNA or advantageous matching properties of the electrical connection. With the wideband properties of the components specified for a RF-frequency of 0.1 to 3 GHz (used here at 0.5 GHz) this PCB enables proton-based imaging in field strength of 3 T and more or even multi-nuclei applications.

Especially, the higher gradient fields can enhance the true resolution of information in the images if it is, in the case of single point imaging, limited by the effects of free diffusion. The true resolution in free water calculated for Figs. 7 and 8 of $\:{\varDelta\:}_{\text{t}\text{r}\text{u}\text{e}}$ = 11.0 μm gives a worst-case scenario. Since the movement of the protons is hindered in the embryos by cell walls and other biological material, the PSFs used to determine Eq. (9) might result narrower. Thus, the spatial resolution may be even better but, still limited by the limited k-space sampling that yields a possible true resolution of $\:{\varDelta\:}_{\text{t}\text{r}\text{u}\text{e}}$ = 10.9 μm (by determination of the FWHM of the sampling introduced PSF^14^).

The feasibility of using gradient amplitudes of up to 9 T/m on MR images was shown (Fig. 6). Higher amplitudes were not possible with a resolution of 10 μm due to the limited gradient rise and fall times. The imaged signal decayed with increasing echo time apparently follows an exponential function. This demonstrates that images benefit from higher gradient amplitudes and indicates that the decay is mainly caused by $\:{T}_{2}^{*}$ and diffusion effects. From the extremely high gradient amplitudes quantitative imaging methods could benefit as well. For example, diffusion imaging where in a Stejskal-Tanner pulsed gradient diffusion sequence the b-values scale quadratically with the gradient amplitude or q-space imaging with shorter gradient pulses.

The introduction of the MOCTI sequence and reconstruction algorithm did successfully generate images of increased CNR compared to the reconstruction with averaging of multiple magnitude images. Averaging of the complex k-space data corresponds to a longer measurement time of a single data point in CTI (decrease of measurement bandwidth) or complex averaging in time domain. The averaging of complex data that would have the potential of a maximum of CNR suffers from signal cancellation due to an inhomogeneous underlaying magnetic field. To overcome this signal voids, the MOCTI reconstruction was designed and demonstrated the potential of the correction with a magnetic field map. For higher amplitudes of the magnetic inhomogeneities or longer echo times, the difference between the algorithms should be even more prominent then demonstrated. The additional inclusion of the navigator for a correction of frequency drifts needs no additional RF-hardware and sample as realized e.g. by Weiger et al.^5^. With an observed frequency drift in the order of 100 Hz over a 47 h scan, the phase during the acquisition of a k-space point (13.6 ms measurement time) can vary by more than 360° compared to a measurement with perfectly adjusted reference frequency. This would cause observable image artifacts, if not corrected.

The high potential of the introduced MRM system was proven by imaging zebrafish embryos. The details in the MRM images match well with the optical microscope image. Features like the eyes and the yolk can easily be identified and compared to the images from the light microscope. Additional features can clearly be differentiated due to the MR specific contrasts and volumetric dimensions can be derived from the three-dimensional image data. Also, the three‐dimensional representations indicate that there are no distortions over the whole measurement volume.

## Conclusion

The suggested microscopy insert has been integrated successfully in a conventional preclinical horizontal bore system and operated with the vendors system control functionality, user interface and gradient amplifiers. No vendor hardware must be removed, and the insert is installed or uninstalled within a few minutes. A transfer to other systems e.g. with bigger bore diameters or different magnetic field strength needs no modifications (provided that a suitable interface to the channels of the gradient amplifiers exists). Even though the acquisition times are still limiting, the insert is well suited for dedicated application in which non-destructive soft-tissue analysis appears relevant. Optimization of e.g. MR contrast or coils may further facilitate broader application of the technique.

## Data Availability

The data supporting this study are available from the corresponding author upon reasonable request.
